# Efficacy of Japanese Maca Powder Against Aging Stress in Human Dermal Fibroblasts

**DOI:** 10.3390/ijms27104471

**Published:** 2026-05-16

**Authors:** Katsuaki Dan, Keita Takanashi, Shinya Kitamura

**Affiliations:** 1 Division of Research and Development, Research Organization of Biological Activity, Tokyo 150-0001, Japan; kitamura0101@gmail.com; 2 Department of Pathophysiology, Yokohama University of Pharmacy, Yokohama 245-0066, Japan; keita.takanashi@yok.hamayaku.ac.jp

**Keywords:** Japanese maca, aging stress, skin cell, ultraviolet radiation, advanced glycation end products, H_2_O_2_, reactive oxygen species

## Abstract

The aim of this study was to identify and examine materials that have a long history of use in folk medicine and exhibit biological activity but have not been fully utilized. This study evaluated the reactivity of Japanese maca powder in cultured human dermal cells subjected to aging stress (UV irradiation, AGE treatment, or H_2_O_2_ treatment). The mRNA levels of three stress parameters (collagen, elastin, and hyaluronic acid synthase) were measured using quantitative reverse transcription polymerase chain reaction. The activity of a prototype Japanese maca powder sample was compared with that of samples subjected to fermentation, room-temperature enzyme, and rapid freeze-drying treatments. Inhibitory effects on reactive oxygen species (ROS) were measured, and the expression of genes involved in senescent cell removal (JAG1) and regeneration promotion (EGF) was examined. Finally, the expression of molecules involved in senescent cell phagocytosis (STAB1) and stem cell phagocytosis signaling and regeneration promotion (FGF2) in macrophages was evaluated. The four types of maca samples altered the mRNA levels of the three stress parameters, conferred resistance to various aging stresses, and delayed suppressed intracellular ROS accumulation. These findings suggest that Japanese maca may help to protect skin cells from age-related stress.

## 1. Introduction

The concept that food and medicine have the same origin is a perspective in modern medicine. Several substances that have been used in folk medicine for many years have not been fully utilized despite exhibiting physiological activity.

**Figure 1:**
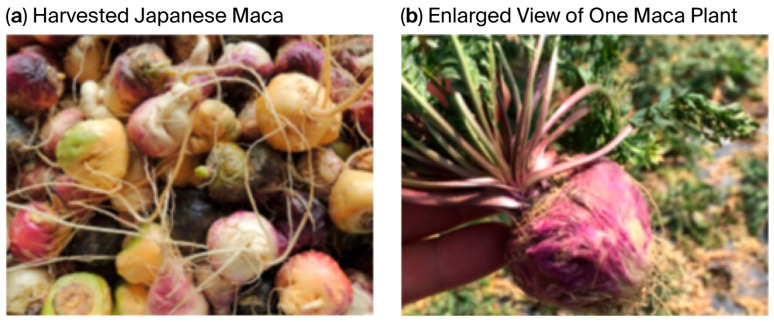
Japanese maca, successfully cultivated in Japan.

Maca (*Lepidium meyenii*) is a perennial plant of the Brassicaceae family, native to Peru in South America. Figure 1 shows an image of maca that has been improved for cultivation in Japanese soil.

Maca roots are used medicinally and have been cultivated as an important food source since the Inca Empire. Maca thrives in harsh natural environments with intense ultraviolet rays, acidic soil, and extreme temperature differences between day and night. High altitudes are ideal for its cultivation; however, because it absorbs nutrients evenly from the soil, land once cultivated with it remains barren for several years. Dried maca roots can be stored for up to seven years, making them a popular option in food preservation. Maca is rich in essential nutrients; 100 g of dried maca contains 59 g of carbohydrates, 10.2 g of protein, 8.5 g of fiber, and 2.2 g of lipids, along with significant amounts of essential amino acids, iron, and calcium. It also contains a large amount of benzyl glucosinolate, which is its main active ingredient (2.2 g) (Table 1).

The biological activities of maca range from energy metabolism and immune regulation to anti-cancer and anti-muscle atrophy effects, as well as libido-enhancing effects [1,2,3,4,5,6]. Furthermore, it is used as hormone replacement therapy for stress-induced premature menopause. Swimming exercise experiments using mice suggested that maca extract exerts effects that improve endurance and reduce fatigue. In addition, in mice given a high-fat diet in combination with maca extract, anti-obesity effects were observed [7,8,9]. The effects of maca extract extend to glucose and lipid metabolism, sleep, and neurological disorders [10,11,12]. There are also reports on clinical data [4]. The effects of the oral intake of maca have been studied for many years; however, its effects on the skin have only recently attracted attention [13].

Natural compounds and extracts are believed to follow the concept of hormesis. Hormesis posits that stimulation occurs at appropriate/moderate doses, while inhibition occurs at high doses [14,15,16,17,18]. While it’s unclear whether Japanese maca follows this concept, the results may offer some indication.

Skin tissue is exposed to various external environmental stressors daily. Skin cells (e.g., keratinocytes, fibroblasts, and dermal stem cells) coordinate their functions to maintain the extracellular matrix environment and sustain stress responses.

The main causes of skin aging include various environmental factors, such as glycation (the accumulation of advanced glycation end products (AGEs)), oxidation (stimulation by hydrogen peroxide), and photoaging (exposure to ultraviolet light). These stressors induce cellular aging, and the accumulation of reactive oxygen species (ROS) within the cells accelerates cellular aging. Therefore, enhancing the resistance to stresses that adversely affect cells is considered a strategy for combating skin aging [19,20].

It is also crucial to eliminate senescent cells and promote the regeneration of new ones. The genes involved in this mechanism have been identified. Reportedly, JAG1 is involved in the elimination of senescent cells in skin tissue, and, after elimination, EGF stimulates the regeneration of surrounding stem cells [21,22,23,24]. STAB1 is also expressed in macrophages to phagocytose senescent cells, and FGF2 stimulates regeneration after phagocytosis [24].

**Figure 2:**
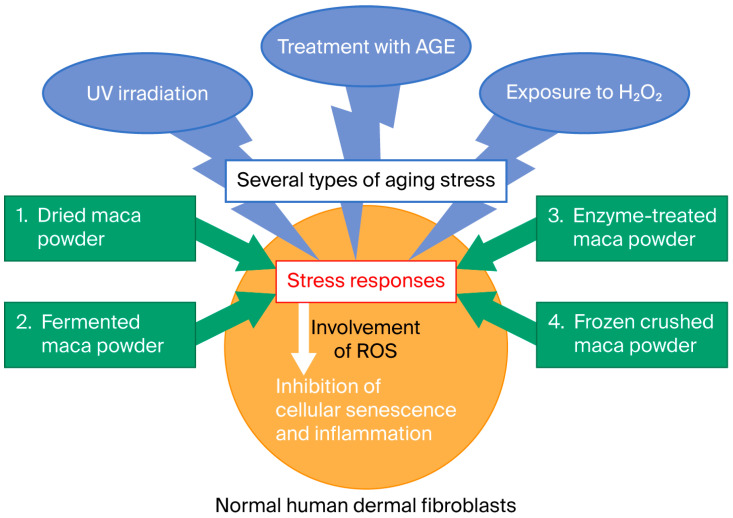
Schematic diagram of the experiment investigating cellular responses to aging stress: Human dermal fibroblasts (HDFs) were subjected to UV irradiation, AGE treatment (glycation), and H_2_O_2_ exposure (oxidation). Four types of maca samples were administered in accordance with these treatments. The cellular stress response was measured based on changes in the expression of three parameters (collagen, elastin, and hyaluronan synthase).

In this study, we investigated the efficacy of Japanese-made maca powder, a variety of maca originally imported from Peru and subsequently modified for cultivation in Japan, against human skin aging. We examined its reactivity in cultured human dermal fibroblasts (HDFs) and its resistance to damage caused by various aging stressors (ultraviolet radiation, glycation stress, AGEs, oxidative stress, and H_2_O_2_). Furthermore, we investigated whether several post-treatments of maca powder (fermentation, room-temperature enzyme, and rapid freeze-drying treatments) would alter its activity. Specifically, we measured the time course of several parameters involved in maintaining skin homeostasis (collagen, elastin, and hyaluronan synthase mRNA levels) [25,26]. Moreover, we examined whether oxidative stress suppresses the accumulation of ROS in cells. Furthermore, we examined the gene expression of molecules involved in the elimination of senescent cells and identified new regeneration mechanisms in skin tissue. A diagram of the study concept is presented in Figure 2.

## 2. Results

### 2.1. Changes in mRNA Levels of Three Stress Parameters in Normal HDFs Following Maca Addition

**Figure 3:**
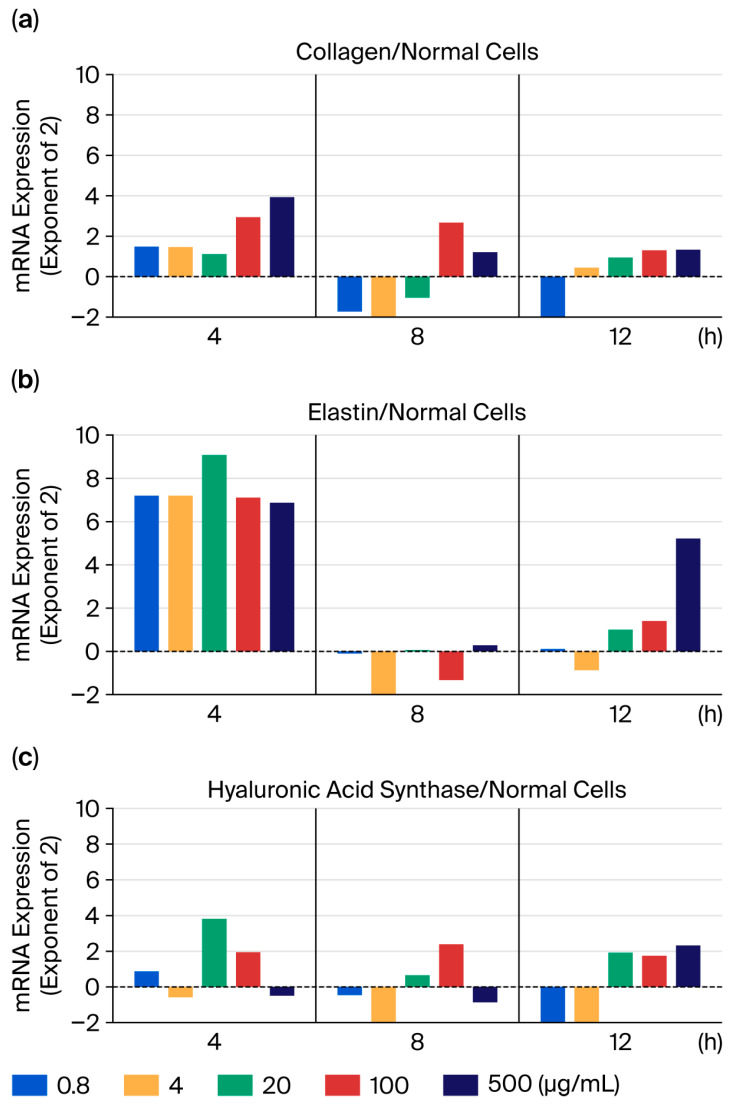
Changes in mRNA expression of three stress parameters in normal HDFs following the addition of dried maca powder (maca sample 1). (**a**) Collagen; (**b**) Elastine; (**c**) Hyaluronic Acid Synthase.

After the addition of maca sample 1, the mRNA levels of collagen, elastin, and hyaluronic acid synthase were measured. Before maca addition, expression levels were set to zero; following maca addition, they increased at each concentration after 4 h and tended to decrease by 8 h. At a high concentration of 500 μg/mL, expression levels increased by 12 h. However, concentration dependence was unclear, and no cytotoxicity was observed, even at 500 μg/mL (Figure 3). However, because the concentration dependence was unclear and a certain effect was observed at 0.8 mg/mL, we decided to continue the study at low concentrations (0.8 and 4 mg/mL) to minimize the number of experiments.

**Figure 4:**
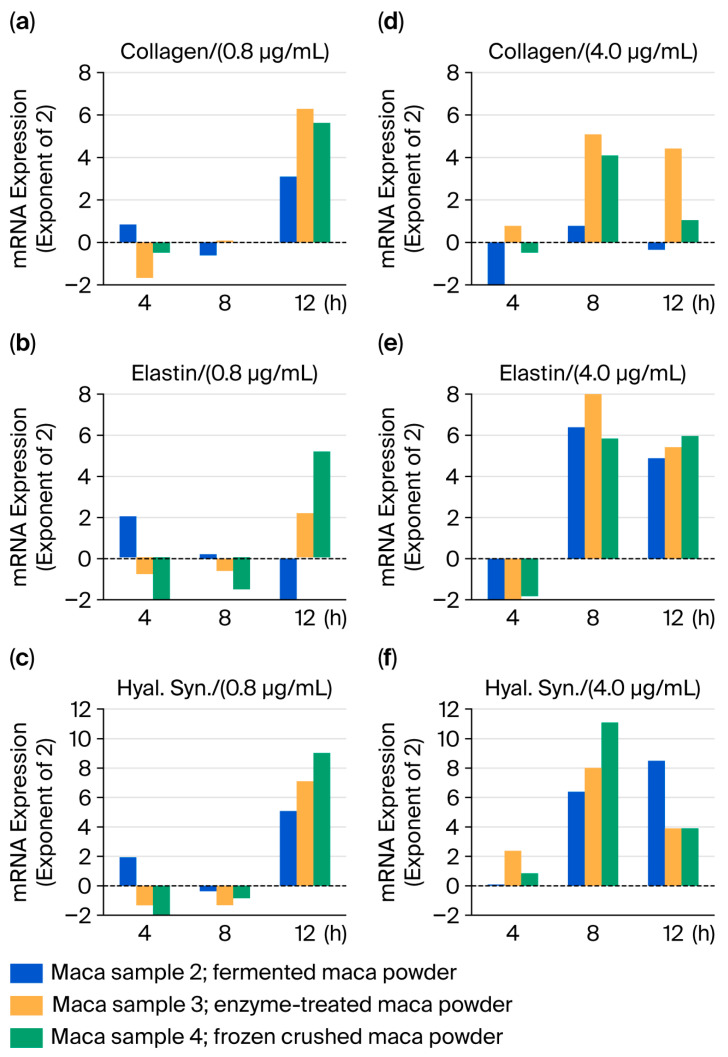
Changes in expression of three stress response parameters (collagen, elastin, and hyaluronan synthase) following the addition of maca samples 2, 3, and 4 to normal HDFs (compared to maca sample 1). (**a**) Collagen/(0.8 μg/mL); (**b**) Elastine/(0.8 μg/mL); (**c**) Hyaluronic acid synthase/(0.8 μg/mL); (**d**) Collagen/(4 μg/mL); (**e**) Elastine/(4 μg/mL); (**f**) Hyaluronic acid synthase/(4 μg/mL).

The mRNA levels of maca sample 1 were compared with those of the other three maca-treated samples (maca samples 2–4). At 0.8 μg/mL, an additive effect was observed at 12 h in all three samples. At 4 μg/mL, an additive effect was observed starting at 8 h. No significant changes were observed in any of the three maca samples subjected to post-treatment (fermentation, enzyme, and rapid freeze-drying treatments) (Figure 4).

### 2.2. Changes in Three Parameters Following Single Application of Three Aging Stresses

**Figure 5:**
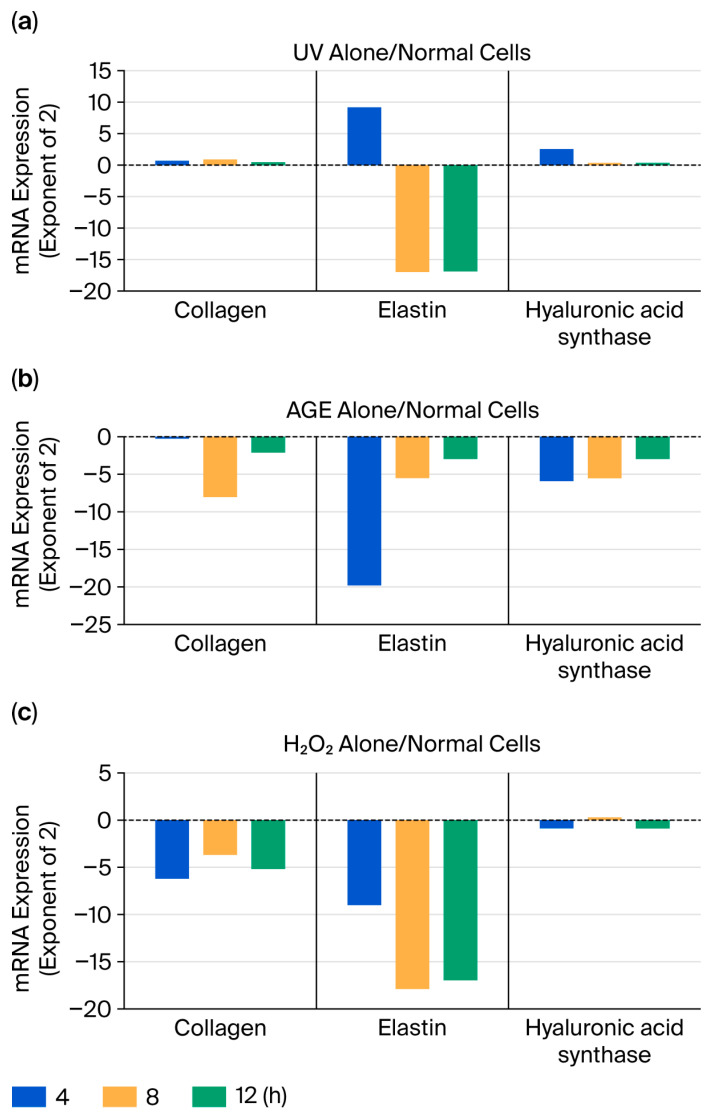
Changes in three parameters (collagen, elastin, and hyaluronan synthase) following single application of three aging stresses (UV irradiation, AGEs, and H_2_O_2_) to normal HDFs. (**a**) UV Alone; (**b**) AGE Alone; (**c**) H_2_O_2_ Alone.

Changes in the three parameters following a single application of the three stresses to HDFs were examined over time. As the results were similar and showed almost no concentration dependency, only the results obtained at a concentration of 4 μg/mL are shown (Figure 5).

Following UV stimulation, elastin showed a transient increase after 4 h, followed by a rapid decrease after 8 h. Both AGE and H_2_O_2_ tended to suppress the three parameters. AGE stimulation induced a transient decrease at 4 or 8 h, followed by a return to steady-state levels. H_2_O_2_ stimulation caused a sustained decrease.

### 2.3. Maca’s Resistance to Photoaging (UV) Stress

**Figure 6:**
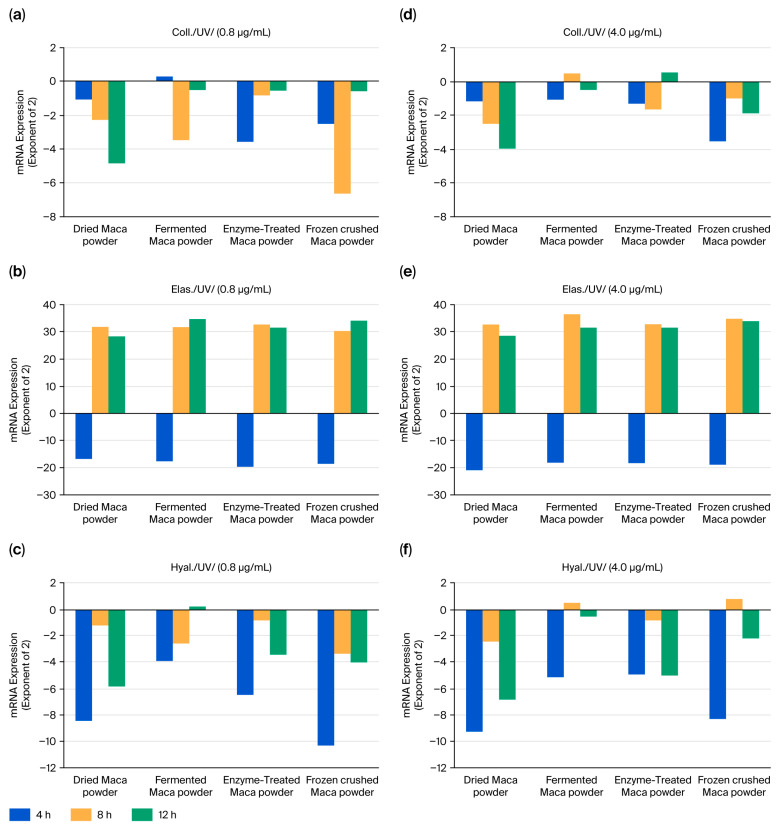
Effect of maca treatment on the expression of collagen (**a**,**d**), elastin (**b**,**e**), and hyaluronan synthase (**c**,**f**) in UV-irradiated HDFs: Differences in mRNA levels of 2 or more compared to the control group were considered significant.

Changes in the parameters following UV stimulation were examined over time and reported as increases or decreases relative to the results of the control group (Figure 6). Similar changes were observed in all four samples at the maca concentrations tested (0.8 and 4 μg/mL). Elastin levels remained elevated after 8 h, whereas the collagen and hyaluronic acid synthase responses tended to be suppressed. Hyaluronic acid synthase responses were inhibited twice, at 4 and 12 h. Almost no differences were observed among the four types of maca samples.

### 2.4. Maca’s Resistance to Glycative Stress (AGEs)

**Figure 7:**
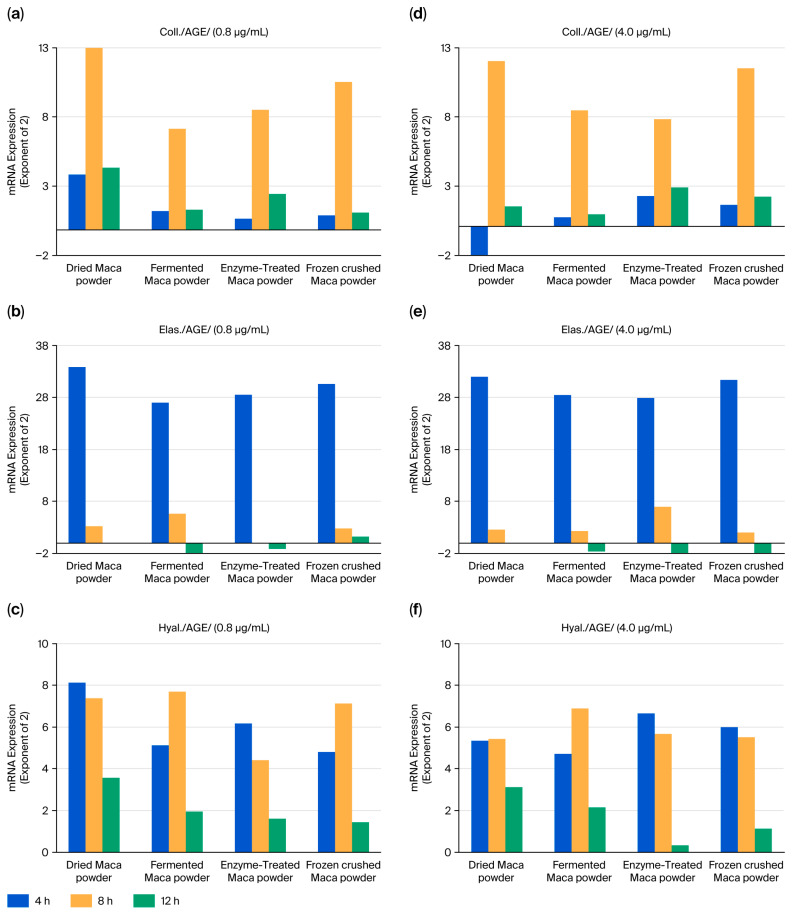
Effects of maca treatment on the expression of collagen (**a**,**d**), elastin (**b**,**e**), and hyaluronan synthase (**c**,**f**) in HDFs under AGE-induced stress conditions. Differences in mRNA levels of 2 or more compared to the control group were considered significant.

The time course of the parameters following AGE stimulation was also examined (Figure 7). All four samples at two concentrations (0.8 and 4 μg/mL) showed similar increases. Collagen and elastin levels increased before 8 h and 4 h, respectively, whereas hyaluronic acid synthase levels remained constant from 4 to 8 h. Almost no differences were observed among the four types of maca samples following AGE stimulation.

### 2.5. Maca’s Resistance to Oxidative Stress (H_2_O_2_)

**Figure 8:**
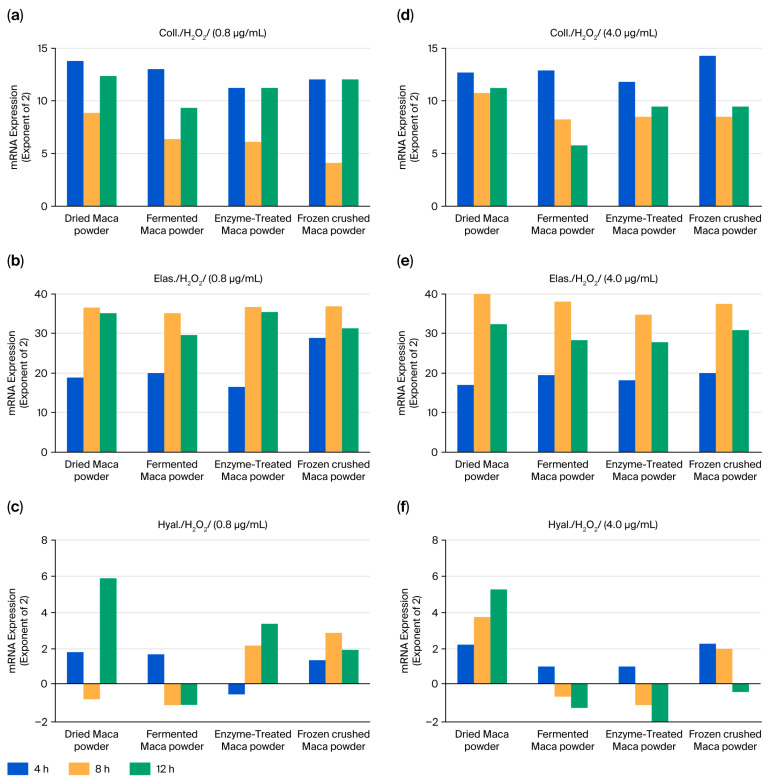
Effects of maca treatment on collagen expression (**a**,**d**), elastin (**b**,**e**), and hyaluronan synthase (**c**,**f**) in HDFs under hydrogen peroxide stress conditions. Differences in mRNA levels greater than the square of two compared to the control group were considered significant.

The time course of these parameters following H_2_O_2_ stimulation was also examined (Figure 8). All parameters tended to increase from 4 h to 12 h and remained elevated; however, the increase in hyaluronic acid synthase levels was small. Concentration had no effect, and the four samples showed similar results.

### 2.6. Maca’s Inhibitory Effect on Intracellular ROS Accumulation and Cell Morphological Changes in Oxidatively Stressed HDFs

**Figure 9:**
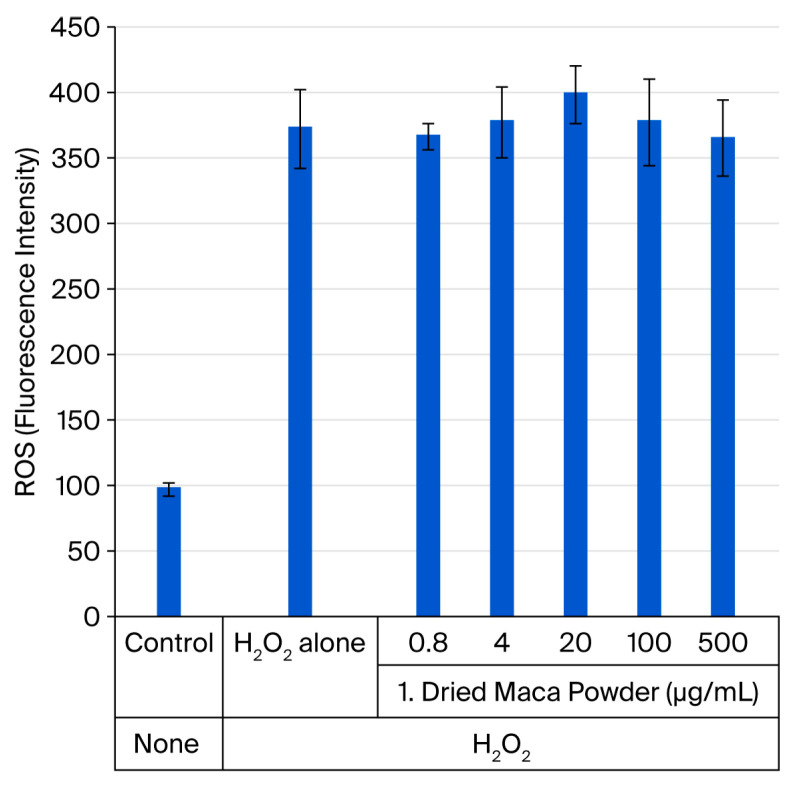
Inhibition of intracellular ROS accumulation in HDFs subjected to oxidative stress (H_2_O_2_) after 12 h of maca treatment.

H_2_O_2_ stress induced ROS accumulation in HDFs, increasing the fluorescence intensity, which reflects intracellular ROS levels, from 95 to 370 in the control. The addition of the four types of maca samples at various concentrations for 12 h under these conditions did not significantly suppress ROS production. As the results were similar across groups, only those for sample 1 are shown (Figure 9).

**Figure 10:**
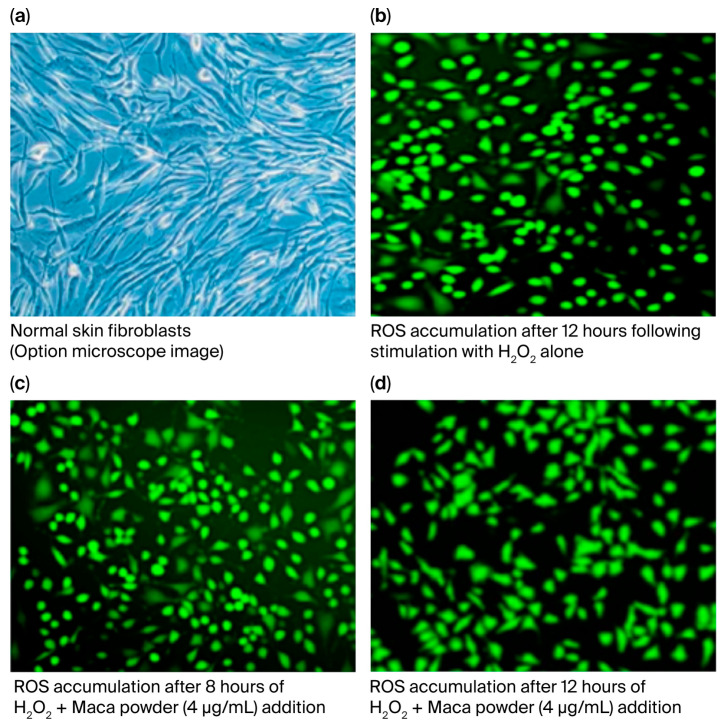
Changes in cell morphology after maca treatment in HDFs subjected to oxidative stress (H_2_O_2_) (**a**): light microscopy image; (**b**–**d**): fluorescence microscopy images).

Figure 10a shows a bright-field image of normal HDFs obtained using an optical microscope. Fibroblasts with fibrous structures were observed. In contrast, when ROS-accumulating cells were observed using fluorescence microscopy, they were found to exhibit a circular, atrophied morphology due to H_2_O_2_ (Figure 10b). However, after 8–12 h of maca addition, an increasing number of cells recovered their original fibrous morphology (Figure 10c,d).

**Figure 11:**
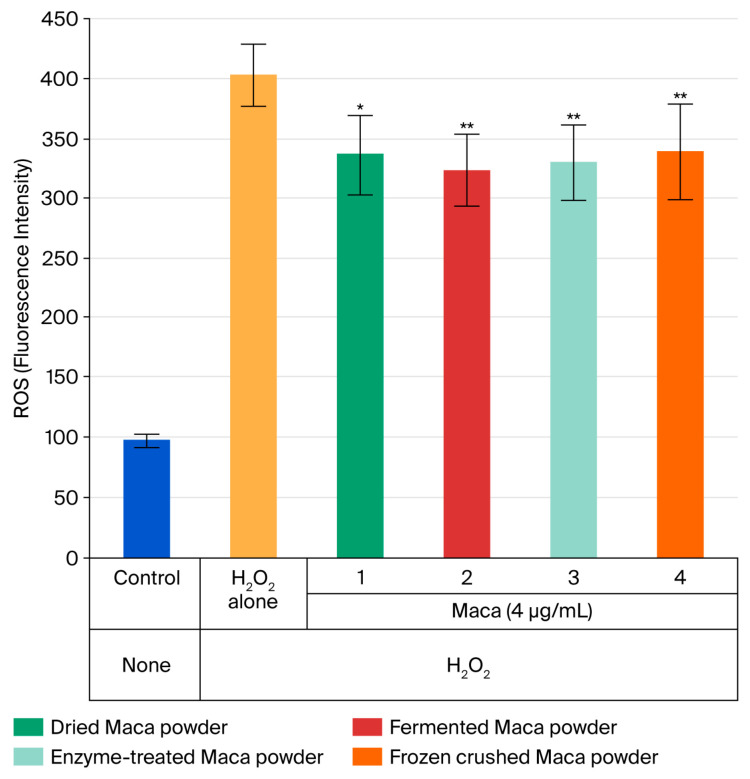
Inhibition of intracellular ROS accumulation in HDFs subjected to oxidative stress (H_2_O_2_) after 24 h of maca treatment. * *p* < 0.05, ** *p* < 0.001.

The accumulation of ROS within cells was observed again after 24 h. H_2_O_2_ stress increased the fluorescence intensity to 403. However, all four samples showed a significant inhibitory effect on ROS accumulation. No significant differences were observed between the four types of samples. In other words, these physiological activities were not lost even after treatment (fermentation, enzyme, and rapid freeze-drying treatments) of the prototype maca samples (Figure 11).

### 2.7. Expression of a Gene That Activates the Senescent Cell Elimination Mechanism (JAG1) and Promotes Regeneration (EGF) in Fibroblasts

**Figure 12:**
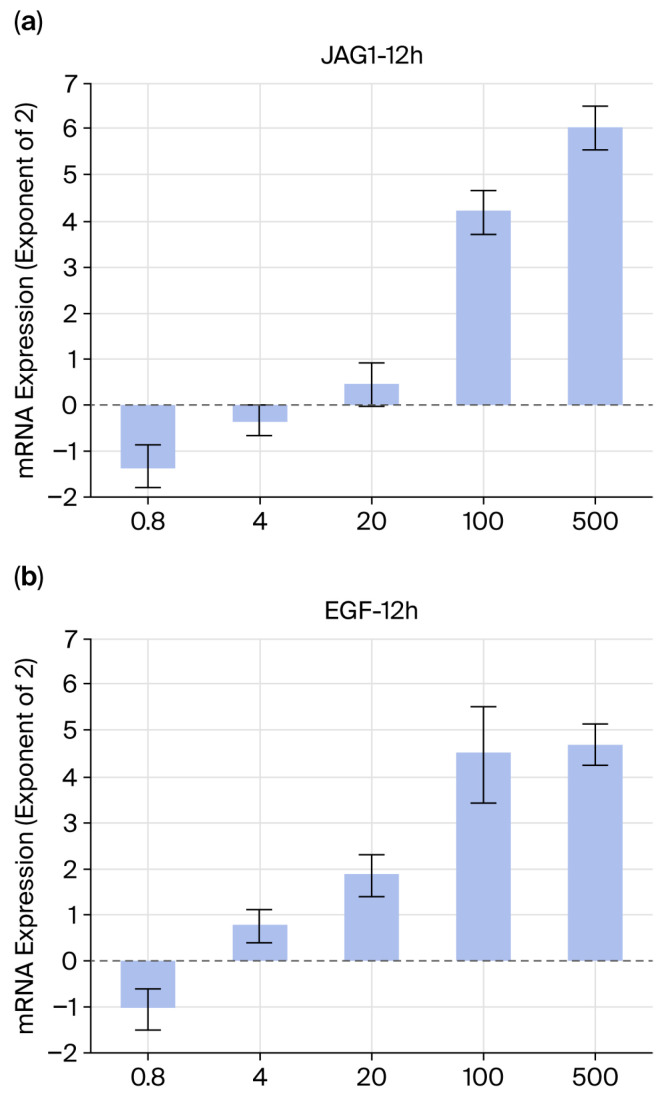
Expression of a gene (JAG1) that activates the senescent cell clearance mechanism and a gene (EGF) that promotes regeneration in HDFs. (**a**) JAG1-12h; (**b**) EGF-12h.

In fibroblasts, 12 h after the addition of maca powder sample 1, the expression of both JAG1 and EGF increased in a concentration-dependent manner, with significant differences observed at concentrations of 100 μg/mL and above (Figure 12).

The expression of a gene that activates senescent cell elimination (STAB1) and promotes regeneration (FGF2) in macrophages was examined.

**Figure 13:**
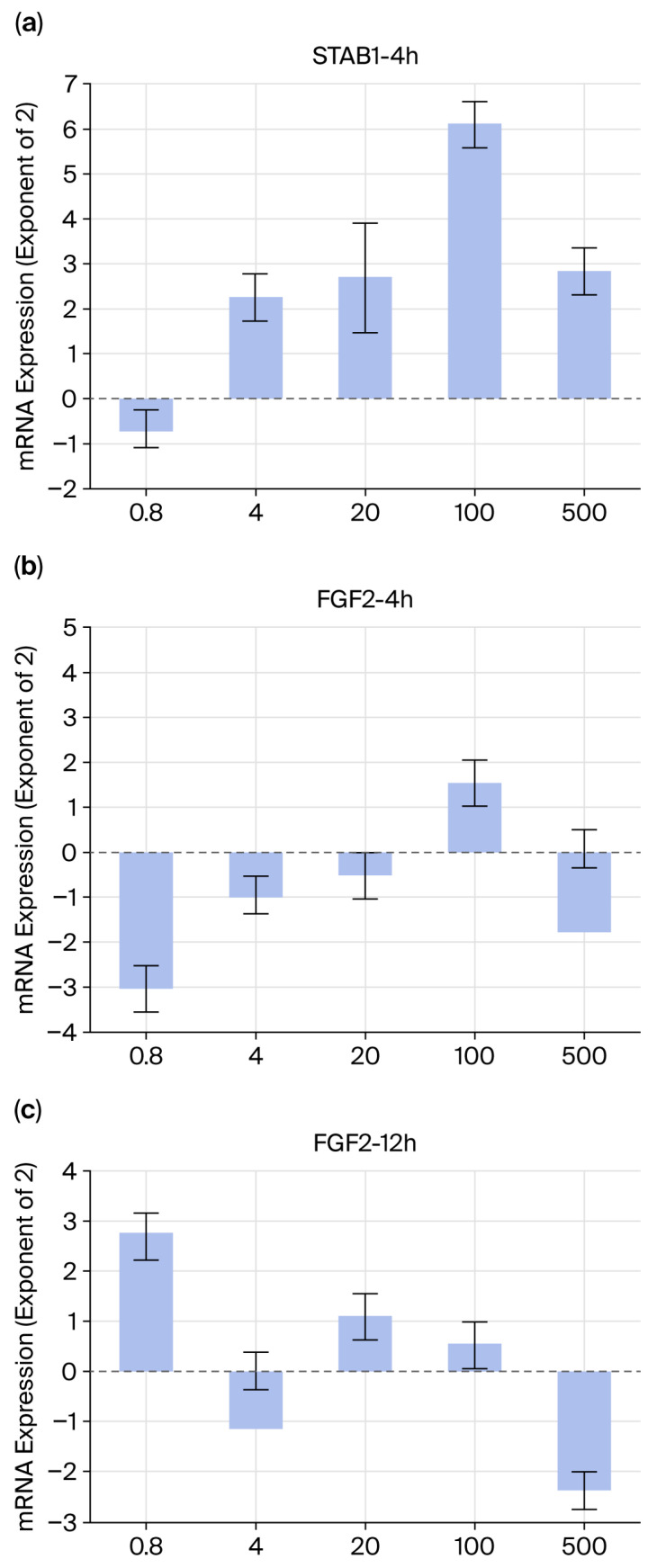
Expression of a gene (STAB1) that activates the senescent cell clearance mechanism and a gene (FGF2) that promotes regeneration by macrophages. (**a**) STAB1-4h; (**b**) FGF2-4h; (**c**) FGF2-12h.

In macrophages, 4 h after the addition of maca sample 1, STAB1 expression significantly increased at concentrations of 4 μg/mL and above (Figure 13a). FGF expression showed a concentration-dependent increase at 4 h; however, although an upward trend was observed at 100 μg/mL, this was not statistically significant (Figure 13b). At 12 h, the concentration dependence was unclear, but an increase was observed even at concentrations as low as 0.8 μg/mL (Figure 13c). Both STAB1 and FGF2 deviated from concentration dependency at 500 μg/mL (Figure 13b,c).

## 3. Discussion

Maca, native to Peru, has a long history of consumption and has undergone extensive testing for its medicinal properties [1,2,3,4,5,6,7,8,9,10,11,12,13]. For this study, we legally imported maca from Peru into Japan, and, using a variety that was selectively bred to suit the Japanese environment, we investigated whether Japanese-grown maca can confer resistance to aging stress, focusing on its use in a wide range of applications beyond conventional oral administration by specifically targeting the skin.

Japanese maca is available in a variety of colors, including black, red, yellow, purple, and gray, and the efficacy of each color has been tested, primarily based on Peruvian maca [4]. Although a diverse range of efficacies has been tested, formulations containing multiple colors have been shown to exhibit a wider range of effectiveness. Furthermore, hybrids of different colors exist, and it appears that clear differentiation is difficult at the cultivation site. We confirmed that the maca used in this study contained black, red, and yellow colors. The discovery of novel active ingredients based on these color differences is an important topic. However, genetic classification is required for more detailed investigations, and this is outside the scope of this study. With a view to social implementation, we believe that some degree of coexistence of multiple maca subtypes is unavoidable, and, therefore, in this study, we used dried maca powder containing all three subtypes as a prototype. The nutritional content and amount of benzyl glucosinolate (the active ingredient) in Japanese maca do not particularly differ from those in Peruvian-produced maca, as shown in Table 1; in fact, the results show higher values.

Our research has primarily focused on the anti-aging effects associated with the regenerative function of the skin. We previously studied the development of cosmetics utilizing the antioxidant properties of antibacterial and antiviral compounds [27] and demonstrated the additive anti-skin aging effects of pig placenta extract and stem cell-derived exosomes [28]. Although these are active synthetic and animal-derived products, the maca used in the present study is a plant-derived powder. We hope to explore the possibility of the future differentiation and application of these materials for their anti-aging effects.

In the present study, we focused on skin anti-aging effects, and we examined the resistance of skin fibroblasts to aging stress using four types of samples, namely, a basic maca powder sample (prototype) and maca samples obtained after post-processing treatments, namely, fermentation, enzyme, and flash-freezing treatments, involving the pulverization of raw maca into a dried powder.

When sample 1 was added to normal cells, elastin levels significantly increased within 4 h, and, at higher concentrations, they tended to increase again after 12 h (Figure 3). These effects were more pronounced in post-treatment samples 2–4 than in sample 1. At 8 and 12 h, the 4 μg/mL concentration exerted stronger effects than the 0.8 μg/mL concentration, and the higher concentrations tended to exert effects earlier (Figure 4).

When the three aging stresses were applied individually, each of the three parameters decreased; however, by 12 h, they tended to return to their original levels (Figure 5). After UV stimulation, elastin levels transiently increased at 4 h but then began to decline. AGE stimulation induced a transient decrease at 4 or 8 h, followed by a tendency to return to steady-state levels. H_2_O_2_ stimulation caused a sustained decrease. Although there was variability depending on the parameters, this may indicate a return to original levels over time, suggesting the maintenance of homeostasis.

UV irradiation suppressed the stress-induced changes in collagen, hyaluronic acid, and synthases (Figure 6). However, AGE and H_2_O_2_ stimulation tended to increase almost all three of these parameters. The effect of AGEs was apparent at 4 and 8 h (Figure 7), and H_2_O_2_ stimulation increased this effect from 4 to 12 h (Figure 8). Although increasing stress-induced mRNA levels is not always beneficial, maca may resist or influence these changes.

We investigated the changes in the three parameters in dermal fibroblasts under three types of aging stress using porcine placenta extract (Pla-Ex) [28]. While the responses to AGEs were similar for all three parameters, the collagen responses to UV and H_2_O_2_ stimulation were almost diametrically opposed. Specifically, UV stimulation increased collagen production in Pla-Ex but decreased it in maca, and H_2_O_2_ stimulation decreased collagen production in Pla-Ex but increased it in maca. Although their origins differ (being derived from animals and plants), both are complex compounds, and these diametrically opposed responses to UV light suggest some significance.

ROS accumulation is an important factor in determining intracellular aging. Initial observations 12 h after maca treatment yielded no significant results (Figure 9). However, we noticed a change in the shape of the cells. This was also confirmed after 12 h (Figure 10b). With the addition of maca, the results showed no difference to those obtained after H_2_O_2_ stimulation alone at 8 h, and the cells were rounded in shape; however, after 12 h, the cell shape was already recovering, and fibrous cells were observed (Figure 10c,d). This was thought to indicate that cell damage was repaired quickly by maca; thus, a similar test was performed on ROS levels after 24 h, and significant suppression of ROS accumulation was observed in all four samples (Figure 11). Maca may enhance or accelerate the body’s ability to maintain homeostasis when intracellular ROS accumulation reaches a certain level due to H_2_O_2_ stimulation.

The molecules involved in the elimination of senescent cells in skin tissue and signaling that promotes regeneration after the elimination of nearby tissue stem cells have been identified [21,22,23,24]. Maca treatment increased the levels of both molecules (JAG1 and EGF) at 12 h (Figure 12). Furthermore, the levels of a molecule (STAB1) that phagocytoses senescent cells into macrophages increased in a concentration-dependent manner after 4 h. However, while there was no clear increase in FGF2, a molecule that signals phagocytosis to stem cells and promotes regeneration, observations at 4 h showed a significant increase even at low concentrations (0.8 μg/mL) at 12 h (Figure 13). This may be because it took time for the macrophages to transmit the phagocytic signal to nearby stem cells or because it took time for FGF2 mRNA levels to increase.

These results suggest that Japanese maca may confer resistance to skin aging stress or support the inherent resistance of skin cells. Furthermore, in both skin cells and macrophages, Japanese maca can affect signaling that eliminates senescent cells and promotes regeneration in nearby stem cells. Future studies are needed to verify whether orally administered maca components are digested and absorbed, enter the bloodstream, and reach skin cells via skin capillaries.

In some experiments, a hormesis-like response may be possible. For example, in Figure 3b, when added to normal cells, elastin levels increased within 4 h, and at higher concentrations, a tendency for further increases was observed after 12 h. In macrophages, STAB1 expression also significantly increased at a concentration of 4 µg/mL 4 h after the addition of maca sample 1 (Figure 13a), while an increase was observed even at a low concentration of 0.8 µg/mL after 12 h (Figure 13c). And then, both STAB1 and FGF2 showed deviations from concentration dependence at 500 μg/mL [29,30]. Furthermore, it has been shown that natural extracts and food nutrients, at appropriate concentrations, exhibit hormetic effects by activating the endogenous antioxidant pathway (Nrf2) in a time-dependent and dose-dependent manner [31]. However, this study focused on the presence or absence of various stress responses in maca, and therefore did not include sufficient research to discuss hormesis. We intend to use this information for future studies.

It has been reported that fermenting maca with lactic acid bacteria suppresses nitric oxide production and achieves anti-inflammatory and melanin synthesis inhibitory effects [32]. When we investigated the effects on aging stress, we found no significant difference between the fermented product and the enzymatic hydrolysate compared to the untreated powder. It is thought that processing maca may result in both gains and losses of effects; thus, further data are needed.

The inability to confirm concentration dependence in each experiment may be attributable to limitations in the manufacturing process. For example, the non-uniform particle size of the dried powder may have resulted in variable solubility, thereby obscuring the concentration dependence of the active ingredients. Alternatively, it may be that the concentration is already excessive, and, thus, it is necessary to conduct tests at even lower concentrations.

Our study is based on the concept of gut–brain–skin correlations, and we hypothesize that orally administered ingredients are digested, absorbed, and circulated throughout the body via the bloodstream, thereby exerting effects on the skin. Notably, it has been proven that the digestive breakdown products of collagen and hyaluronic acid act on skin tissue to protect against UV damage [33,34]. The digestive breakdown products of maca sample 3 were also found to have some effect on skin cells.

This study focused on the direct effects on skin cells; however, in the future, we aim to examine the gut–brain–skin axis, that is, the possibility that orally administered maca components reach skin cells via the bloodstream and capillaries. In fact, maca has been shown to have effects in neurological disorders [12] and potential neuroprotective effects in ovariectomized rats [35]. Furthermore, as the gut–brain–liver axis has been demonstrated in [36], we would like to investigate the relationship between maca’s influence on the gut environment, the transmission of maca compounds to the liver and their aggregation in the liver, the transmission of information to the brain, and the subsequent promotion of regulatory T-cell activity in the gut, thereby regulating the gut environment. Going forward, we plan to continue testing, focusing first on identifying anti-aging effects, particularly those unique to Japanese-grown maca, while also considering its effects on the liver and brain.

## 4. Materials and Methods

### 4.1. Materials

#### 4.1.1. Japanese Maca

The product’s main ingredient is “Japanese maca,” based on a variety of Peruvian maca seeds purchased from Peru for promotional purposes at the 1990 Osaka World Expo. The seeds were subsequently modified for growth in Japanese soil. To eliminate variations due to different production areas, we blended products harvested from eight locations across Japan (Kagoshima, Oita, Fukuoka, Tokushima, Hyogo, Shizuoka, Nagano, and Saitama) from the end of March to mid-April to ensure consistency.

Maca can be divided into several different colors depending on the cultivation method, including black, yellow, red, and purple. These varieties were blended in specific ratios and dried to a powder called “Japanese maca powder,” which was provided by Maca Japan (Tokyo, Japan). This “Japanese maca powder” was designated as sample 1 (lot 250510) and used as the prototype maca. A fermented liquid was prepared as sample 2 (lot 240724), a dried powder that had undergone room-temperature enzyme treatment was designated as sample 3 (lot 250312), and freeze-dried raw maca powder was designated as sample 4 (lot 202511). Each sample was serially diluted (0.8, 4, 20, 100, and 500 μg/mL) for use in the experiment.

#### 4.1.2. Cells

HDFs were purchased from PromoCell (Heidelberg, Germany) and subcultured in the designated medium at 37 °C in a 5% CO_2_ incubator.

Macrophages were differentiated from purchased monocytes and used in the experiments. The human monocytic cell line U937 was purchased from the European Certified Cell Culture Collection (Wiltshire, UK) and cultured in RPMI-1640 medium containing l-glutamine and phenol red (Fujifilm Wako Pure Chemical Corporation, Osaka, Japan) supplemented with 10% fetal bovine serum (Moregate BioTech, Queensland, Australia), penicillin (100 U/mL), and streptomycin (100 μg/mL) at 37 °C in a humidified atmosphere of 5% CO_2_ and 95% air. These cells were cultured with 25 ng/mL phorbol 12-myristate 13-acetate (PMA) (Sigma-Aldrich Japan, Tokyo, Japan) for 72 h to induce differentiation into macrophage-like cells. The cells were then cultured in a PMA-free medium for 48 h. The differentiated macrophages were transformed into adherent cells. These methods are based on those in previous reports [37,38].

### 4.2. Methods

**Figure 14:**
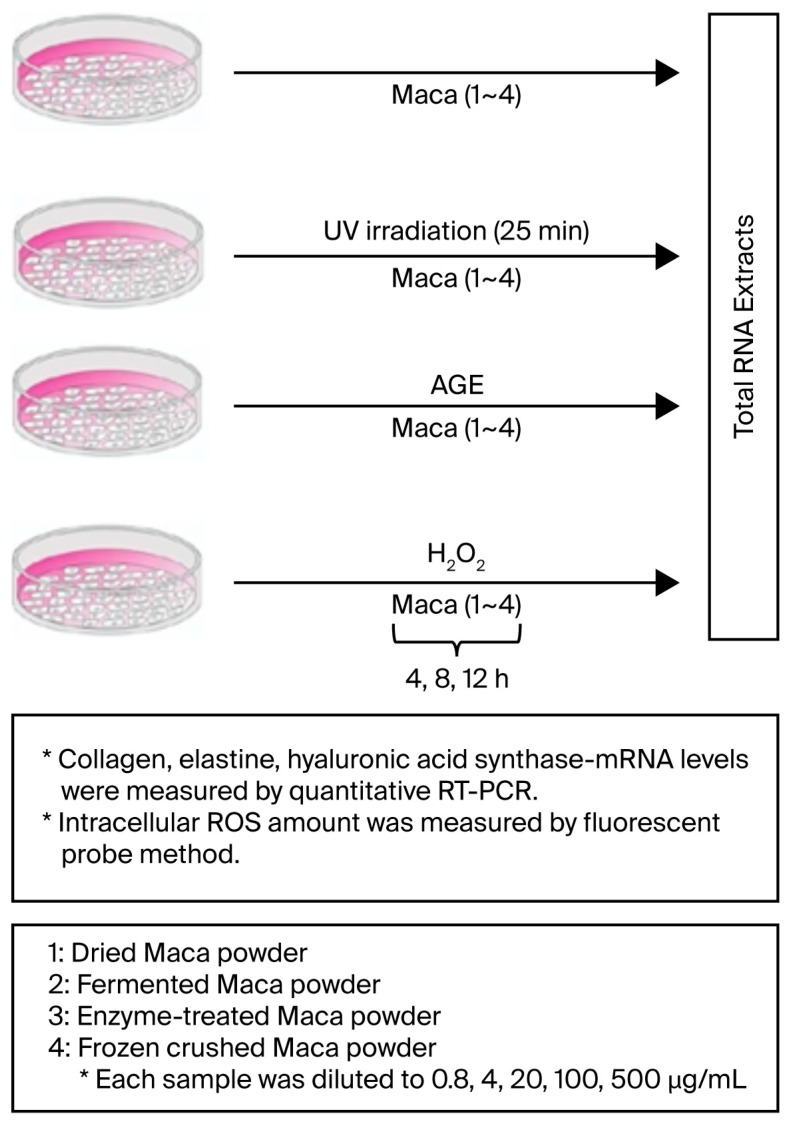
Basic experimental protocol: Maca samples (four types) were diluted five times and treated with HDFs in accordance with aging stress (UV irradiation, AGE, and H_2_O_2_). Total RNA was extracted at each time point (4, 8, and 12 h), and changes in the expression of three parameters of cellular stress response (collagen, elastin, and hyaluronan synthase) were measured using quantitative RT-PCR. Intracellular ROS levels were also measured using a fluorescent probe method.

An outline of the experimental protocol is shown in Figure 14.

Cultured HDFs were subjected to three aging stressors (UV, AGE, and H_2_O_2_). Serial dilutions of maca samples were then added to the cultures at final concentrations of 0.8, 4.0, 20, 100, and 500 μg/mL. Cells were harvested at 4, 8, and 12 h after addition, and intracellular total RNA was analyzed for three mRNAs (collagen, elastin, and hyaluronic acid synthase) using quantitative RT-qPCR. Intracellular ROS levels were also measured in some samples.

#### 4.2.1. Photoaging (UV Irradiation) Stimulation

All wells in a 24-well culture plate containing various cell types were exposed to UV light at a constant distance from the lamp for a set period of time (5 or 25 min), after which the plate was immediately returned to a 37 °C CO_2_ incubator for continued culture. The UV irradiation dose was measured using a UV intensity meter at 2 J/cm^2^ for 5 min and 10 J/cm^2^ for 25 min. After UV irradiation (25 min), the maca was treated, and total RNA was extracted at each time point.

#### 4.2.2. Advanced Glycation End Products (AGEs): Preparation and Stimulation

Bovine serum albumin (BSA Fraction V, Sigma-Aldrich Japan, Tokyo, Japan) was used to treat the AGEs according to the method described by Maeda et al. [21]. BSA (25 mg/mL) was incubated in 0.2 M phosphate buffer (pH 7.4) for 7 days. Subsequently, unbound glyceraldehyde was removed by applying it to a PD-10 gel filtration column equilibrated with phosphate-buffered saline. The amount of glycated BSA in the resulting samples was quantified using a glyceraldehyde-derived AGE ELISA kit.

#### 4.2.3. Oxidative Stress Load

H_2_O_2_ (Wako, Osaka, Japan) was diluted with purified water to a final concentration of 0.2 mM and added to the culture medium [23].

#### 4.2.4. Intracellular Total RNA Extraction

Total intracellular RNA was extracted using TRIzol reagent (Thermo Fisher Scientific K.K. Tokyo, Japan) according to the manufacturer’s protocol.

#### 4.2.5. qRT-PCR

The mRNA expression levels of collagen [39], elastin [40], hyaluronic acid synthase [39], JAG1 [NM000214.3], EGF [NM001963], STAB1 [41], and FGF2 [42] were measured via one-step qRT-PCR using specific primers for each gene (Table 2). One-step PCR, which can be performed in one tube, was performed using a Luna Universal One-Step qRT-PCR Kit (New England Biolabs Japan, Tokyo, Japan). The PCR equipment consisted of a Thermal Cycler Dice Real-Time System II (Takara Bio, Kusatsu, Shiga, Japan). The reactions were performed according to the manufacturer’s protocol. Delta Ct values were calculated using the regulating gene glyceraldehyde-3-phosphate dehydrogenase (GAPDH) as an internal standard [39]. The difference between each experimental and control group was calculated as delta-delta Ct values and is expressed as a fold increase in mRNA expression.

#### 4.2.6. Measurement of Intracellular ROS

Intracellular ROS production was measured fluorometrically using dichlorofluorescein diacetate (DCF-DA, Thermo Fisher Scientific K.K. Tokyo, Japan). Cells from each experimental group were labeled by incubation with 10 mM DCF-DA at 37 °C for 20 min. The fluorescence intensity at an excitation wavelength of 525 nm was measured using an Agilent microplate reader (SYNERGY/BioTek, Santa Clara, CA, USA) to assess intracellular ROS levels. Cell morphology was observed using a fluorescence microscope.

#### 4.2.7. Statistical Analysis

All experiments were conducted with three samples, and the values are presented as the mean ± standard deviation. Statistical analysis was performed using Student’s *t*-test, with *p* < 0.05 considered statistically significant. For qRT-PCR, a difference of two or more from the delta delta Ct value (a difference of 4-fold or more at the mRNA level) was considered statistically significant.

## 5. Conclusions

Japanese maca, used as a dried powder, induced changes in the mRNA levels of collagen, elastin, and hyaluronic acid synthase in skin fibroblasts, indicating that it conferred resistance to aging stress (UV, AGE, and H_2_O_2_), regardless of whether or what type of post-treatment was applied. The onset and duration of this effect varied, and concentration dependence was unclear; however, it can be stated that an effect was observed, even at a low concentration of 0.8 mg/mL. Further investigation at lower concentrations or for longer periods is needed. Although intracellular ROS suppression was not significant after 12 h, cell morphology recovered, and significant and concentration-dependent ROS suppression was confirmed in the 24 h test. These results suggest that Japanese maca may confer resistance to skin aging stress.

## Figures and Tables

**Table 1:** Analysis of Japanese maca components.

	Components	Japanese Maca Powder
Nutrition (per 100 g)	Water	4.2 g
	Protein	20 g
	Fat	2.0 g
	Carbohydrates (1 + 2)	68.2 g
	Dietary fiber (1)	21 g
	Sugars (2)	47 g
	Ash	5.6 g
Minerals (per 100 g)	Na	39.7 mg
	K	
	Ca	312.0 mg
	Mg	94.3 mg
	Fe	3.26 mg
	Zn	2.53 mg
	P	397 mg
	Cu	0.67 mg
Amino acids (per 100 g)	Aspartic acid	1050 mg
	Threonine	460 mg
	Serine	470 mg
	Asparagine	
	Glutamic acid	2200 mg
	Glutamine	
	Glycine	360 mg
	Alanine	460 mg
	Valine	560 mg
	Cysteine	160 mg
	Methionine	120 mg
	Isoleucine	320 mg
	Leucine	450 mg
	Tyrosine	230 mg
	Phenylalanine	310 mg
	GABA	262 mg
	Histidine	290 mg
Active ingredients (per 100 g)	Benzyl glucosinolate	2200 mg

Analyzed by the Japan Food Analysis Center.

**Table 2:** Primer sequences used for one-step qRT-PCR analysis of target genes.

Gene	Primer	References
Collagen1A2	Forward: CTGGACCTCCAGGTGTAAGC	[39]
	Reverse:TGGCTGAGTCTCAAGTCACG	
Elastin	Forward:GGCCATTCCTGGTGGAGTTCC	[40]
	Reverse:AACTGGCTTAAGAGGTTTGCCTCCA	
Hyaluronic acid synthase	Forward:CACGTAACGCAATTGGTCTTGTCC	[39]
	Reverse:CCAGTGCTCTGAAGGCTGTGTAC	
jagged 1 (JAG1)	Forward:TGCTACAACCGTGCCAGTGACT	[NM000214.3]
	Reverse:TCAGGTGTGTCGTTGGAAGCCA	*Locus ID: 182
EGF	Forward:TGCGATGCCAAGCAGTCTGTGA	[NM001963]
	Reverse:GCATAGCCCAATCTGAGAACCAC	*Locus ID: 1950
STAB1	Forward:GAACCATGTGCCACTGGAAGGC	[41]
	Reverse:AGCGGAATCTCCTGGTGCAGTT	
FGF2	Forward:ACTTGGAGGCTTATCACCTGT	[42]
	Reverse:CCAGCATTTCGGTGTTGAAGA	
GAPDH	Forward:GACATGCCGCCTGGAGAAAC	[39]
	Reverse:AGCCCAGGATGCCCTTTAGT	

*Locus ID: OriGene Technologies Inc.

## Data Availability

The original contributions presented in this study are included in the article. Further inquiries can be directed to the corresponding author.

## Abbreviations

The following abbreviations are used in this manuscript:

HDF	Human Dermal Fibroblast
AGE	Advanced Glycation End Product
ROS	Reactive Oxygen Species
EGF	Epidermal Growth Factor
STAB1	Stabilin-1
BSA	Bovine Serum Albumin
GAPDH	Glyceraldehyde-3-Phosphate Dehydrogenase
DCF-DA	Dichlorofluorescein Diacetate
PMA	Phorbol 12-Myristate 13-Acetate
ELISA	Enzyme-Linked Immunosorbent Assay
